# Markers and mechanisms of death in *Drosophila*

**DOI:** 10.3389/fragi.2023.1292040

**Published:** 2023-12-12

**Authors:** John Tower

**Affiliations:** Molecular and Computational Biology Section, Department of Biological Sciences, University of Southern California, Los Angeles, CA, United States

**Keywords:** aging, biomarker, falls, death dance, intestinal barrier integrity, cause of death, eye aging, video assay

## Abstract

Parameters correlated with age and mortality in *Drosophila melanogaster* include decreased negative geotaxis and centrophobism behaviors, decreased climbing and walking speed, and darkened pigments in oenocytes and eye. Cessation of egg laying predicts death within approximately 5 days. Endogenous green fluorescence in eye and body increases hours prior to death. Many flies exhibit erratic movement hours before death, often leading to falls. Loss of intestinal barrier integrity (IBI) is assayed by feeding blue dye (“Smurf” phenotype), and Smurf flies typically die within 0–48 h. Some studies report most flies exhibit Smurf, whereas multiple groups report most flies die without exhibiting Smurf. Transgenic reporters containing heat shock gene promoters and innate immune response gene promoters progressively increase expression with age, and partly predict remaining life span. Innate immune reporters increase with age in every fly, prior to any Smurf phenotype, in presence or absence of antibiotics. Many flies die on their side or supine (on their back) position. The data suggest three mechanisms for death of *Drosophila*. One is loss of IBI, as revealed by Smurf assay. The second is nervous system malfunction, leading to erratic behavior, locomotor malfunction, and falls. The aged fly is often unable to right itself after a fall to a side-ways or supine position, leading to inability to access the food and subsequent dehydration/starvation. Finally, some flies die upright without Smurf phenotype, suggesting a possible third mechanism. The frequency of these mechanisms varies between strains and culture conditions, which may affect efficacy of life span interventions.

## 1 Introduction

Whereas there is no universally accepted definition of aging, one commonly accepted operational definition of aging is a progressive decline in intrinsic physiological function, leading to an age-dependent decrease in rates of survival and reproduction (Flatt, 2012). The mechanism(s) for decline in physiological function leading to increased death rate remain unclear. One way to address this question is to determine cause(s) of death, and investigate whether these causes of death might result from common or unique declines in physiological function. In 2021, the top 10 causes of death in United States of America were #1 heart disease, #2 cancer, #3 COVID-19, #4 unintentional injuries, #5 stroke, #6 lung disease, #7 Alzheimer disease (AD), #8 diabetes, #9 liver disease, and #10 kidney disease (Xu et al., 2022), and each of these causes has age as a major risk factor. *Drosophila melanogaster* is a major research organism for study of aging, due to its short life span and tractable genetics and molecular biology (Tower, 2019; Clancy et al., 2022). Recent studies provide insight into markers and mechanisms of death in *Drosophila*, and their potential similarities, or lack thereof, to causes of death in humans. Analysis of death mechanisms in *Drosophila* may provide insight into underlying aging mechanisms in *Drosophila* and other organisms.

## 2 Parameters that correlate with increased age and rate of death

Multiple behavioral, physiological and molecular parameters have been identified that change with age in *Drosophila*. Parameters assayed in groups of flies can show correlation with age, whereas assay of individual flies is required to demonstrate the ability of a parameter to predict death. Correlated parameters include negative geotaxis behavior (climbing away from gravity), centrophobism behavior (preferential exploration of the outer edges of the container), and walking speed, each of which decreases with age in males and females (Grotewiel et al., 2005; Jahn et al., 2011; Jones and Grotewiel, 2011; Tower et al., 2019). The decreased walking speed is reported to be a major driver of the decreased negative geotaxis behavior (Rhodenizer et al., 2008). Impairment of negative geotaxis behavior with age is accelerated in *Drosophila* models of Parkinson’s disease (PD) (Riemensperger et al., 2013) and AD (Chakraborty et al., 2011; Jahn et al., 2011; Mhatre et al., 2014), and correlates with reduced life span. Finally, blocking apoptosis in ensheathing glia in the brain improved maintenance of negative geotaxis behavior with age, and slightly increased life span (Sheng et al., 2023). These studies are consistent with a causal role for neurodegeneration in the age-associated loss of negative geotaxis behavior, similar to the #7 AD cause of death in humans.

Another parameter that correlates with age in males and females is accumulation of brown age pigment in the oenocytes, which are highly metabolically active liver-like cells containing numerous mitochondria (Makki et al., 2014; Tower et al., 2014; Huang et al., 2019). Studies in mammals implicate incomplete lysosomal degradation of damaged mitochondria in the generation of age pigments (Gray and Woulfe, 2005). Pigments in the male and female *Drosophila* eye also become darker with age (Tower et al., 2014; Tower et al., 2019). Markers of cell death, including increased caspase activity and nuclear DNA fragmentation, increase with age in muscle and fat-body tissue (Zheng et al., 2005). Finally, *Drosophila* heart function deteriorates with age, including systolic and diastolic dysfunction and increased arrhythmia (Blice-Baum et al., 2019), similar to the #1 heart disease cause of death in humans listed above; however, unlike humans, continued regular pumping of the *Drosophila* heart is not required for survival.

## 3 Markers occurring proximal to death

Several changes and phenotypes have been observed to occur in individual flies in the days or hours immediately prior to death.

### 3.1 Egg laying cessation

Egg laying progressively decreases with age, and its cessation is predictive of death of an individual female within approximately 5 days. Rauser et al., 2005 reported egg laying declined rapidly in the 5 days prior to death, with most, but not all, flies declining to zero eggs within 1–3 days prior to death (Rauser et al., 2005). Rogina et al. (2007) also reported rapid decline in the 5 days prior to death, with an average of 0.2 eggs at 2 days prior to death, and zero eggs at 1 day prior to death. Curtsinger compared data from two inbred laboratory strains, four outbred laboratory strains, and three recently collected wild-type stocks (Curtsinger, 2020). The average number of days of survival for individual flies after the last egg ranged from 2.4 days for one inbred strain, to 9.5 days for one wild-type stock, with an average over all strains of 4.7 days.

### 3.2 Eye and body fluorescence changes

Green autofluorescence in the eye and body of male and female *Drosophila* increases several hours prior to death, and continues to increase after death (Tower et al., 2019). Similarly, when GFP expression is targeted to the retinal tissue of adult male and female flies, GFP fluorescence increases several hours prior to death, and continues to increase after death ((Tower et al., 2019); see video Clip 1 and Clip 2). In *C. elegans* and mammals, cell death has been associated with increased green autofluorescence resulting from altered subcellular localization and redox-sensitive changes in the fluorescence of flavins (Levitt et al., 2006; Pincus et al., 2016). Retinal cell death is therefore one candidate for the origin of the increased green autofluorescence observed prior to death of the fly. Indeed, progressive retinal degeneration and photoreceptor cell death during aging are particularly apparent in the *w[1118]* reference strain, which lacks normal eye pigments (Ferreiro et al., 2017). The mechanism for the increased fluorescence of GFP targeted to the retina is currently unclear, but conceivably could be due to alterations in subcellular localization associated with retinal cell death. The increased green autofluorescence and transgenic GFP fluorescence in the *Drosophila* retina has not yet been specifically correlated with any independent markers of cell physiology or cell death, and this may be an useful area for future research.

Consistent with the ability of *Drosophila* eye fluorescence changes to predict imminent death, the eye has been reported to be a diet-sensitive regulator of life span. When *w[1118]* strain flies were maintained on a low-yeast diet, strong visible light shortened life span, and this effect was absent on a high-yeast diet (Shen et al., 2019). In turn, constant darkness extended life span on a high-yeast diet, and these effects were absent on a low-yeast diet (Hodge et al., 2022). Because the high yeast protein diet decreases life span, one possibility is that the negative effects of strong visible light and high yeast protein act through the same pathway (Shen et al., 2019).

Notably, changes in the optical and structural properties of the eye have emerged as promising predictive biomarkers of physiological age, neurodegenerative disease progression, and risk of death in male and female humans. Drusen is an autofluorescent pigment containing proteins derived from blood and retinal epithelial cell debris (Bergen et al., 2019). Fluorescence imaging of the human eye reveals increased drusen abundance in the retina of AD patients (Ashok et al., 2020; Singh and Verma, 2020). Optical coherence tomography reveals thinning of the retinal nerve fiber layer and macular ganglion cell layer in patients with reduced cognitive function and AD (Kesler et al., 2011; Ward et al., 2020). Finally, machine learning models based on images of the fundus and retina provided a predictive biomarker for mortality risk (Ahadi et al., 2023; Zhu et al., 2023). In summary, increased autofluorescence in the retina has emerged as a predictive biomarker of death in *Drosophila*, and as a marker of the #7 AD cause of death in humans, consistent with the idea that neurodegeneration may be a cause of death in *Drosophila* as well as in humans.

### 3.3 Loss of rhythmic behavior and spikes in circadian gene expression

One way that *Drosophila* activity rhythms can be assayed is by using activity monitors, where fly activity is measured by the frequency at which the movement of the fly disrupts an infrared light beam. Activity monitors revealed periods of increased arrhythmic behavior prior to death in *Drosophila* (Levine et al., 2002). Both male and female *Drosophila* exhibit reduced strength of sleep:wake cycles and more fragmented sleep with age, similar to changes observed in humans (Koh et al., 2006). Zhao et al. examined male flies containing transgenic luciferase reporters for the circadian regulatory genes *timeless* and *period* (Zhao et al., 2018). They found that whereas reporter expression declined with age, as expected, there was a surprising spike in expression that lasted approximately 3–4 days, and was centered approximately 3–4 days prior to death. These flies also became completely arrhythmic over a period of 3.7 ± 0.1 days before death, and therefore the spike in gene expression corresponded to a final loss of rhythmic movement. Null mutation of *period* caused reduced negative geotaxis behavior, neurodegeneration and shortened life span, consistent with a possible causative role for loss of rhythmic function in *Drosophila* death (Krishnan et al., 2009). Notably, disruptions in circadian rhythms are causally implicated in the #1 heart disease, #2 cancer, and #7 AD causes of death in humans (Chellappa et al., 2019; Olejniczak et al., 2023).

### 3.4 Bouts of abnormal behavior and falls to supine position

There have long been anecdotal reports in the *Drosophila* research community that flies exhibit periods of abnormal behavior prior to death, sometimes referred to as a “death dance”. Video analysis has proven to be a powerful method for assay of *Drosophila* behavior, aging and death (Zou et al., 2011). In an elegant study, Gaitanidis et al. performed longitudinal analysis of over 1,000 individual male and female flies using video recordings (Gaitanidis et al., 2019). They found two general trajectories for the deterioration of motor function with age. Some flies became progressively incapacitated over several days before death (termed “illderlies”), whereas others became incapacitated only several hours before death (termed “wellderlies”). Both trajectories were observed to converge on a terminal state with stereotypical signs of functional collapse followed by death within ∼3 h. Common features observed included decreased walking and climbing speed, leg joint defects (leg permanently extended or retracted; see Movie 2), climbing defects including falls to a supine position, and a terminal phase typically associated with permanent supine position (see Movie 3). Notably, many flies, in particular among the “wellderlies”, exhibited bouts of increased locomotor activity that lasted up to 2 h, and peaked about 5 h prior to death. These bouts involved increased walking behavior, increased climbing efforts often associated with falls, seizure-like events, and “paradoxical” behaviors including rearing up with front-leg boxing movements followed by a fall to supine position (see Movie 1). No significant sex differences in terminal behaviors were observed. Supine behavior is also predictive of impending death in male medflies (*Ceratitis capitata*), where temporary supine behavior began on average 16 days prior to death, and time spent in supine position increased exponentially until death (Papadopoulos et al., 2002).

Tower et al. used two synchronized video cameras to track male and female *Drosophila* movement through 3D space (Tower et al., 2019). Several flies were analyzed as they died from normal aging, and several young flies were placed in empty vials, and tracked as they died from dehydration/starvation. Tracking was conducted using either reflected visible light, reflected infrared light, or fly fluorescence using flies containing fluorescent transgenic reporter constructs. Old flies exhibited reduced total movement, negative geotaxis and centrophobism behaviors relative to young flies, as expected. The frequency of directional heading changes (FDHC) was calculated to measure erratic movement. Notably, the majority of the flies exhibited one or more bouts of erratic movement 0–8 h prior to death, as indicated by spikes in FDHC, and sometimes leading to falls to supine position (Tower et al., 2019).

**FIGURE 1:**
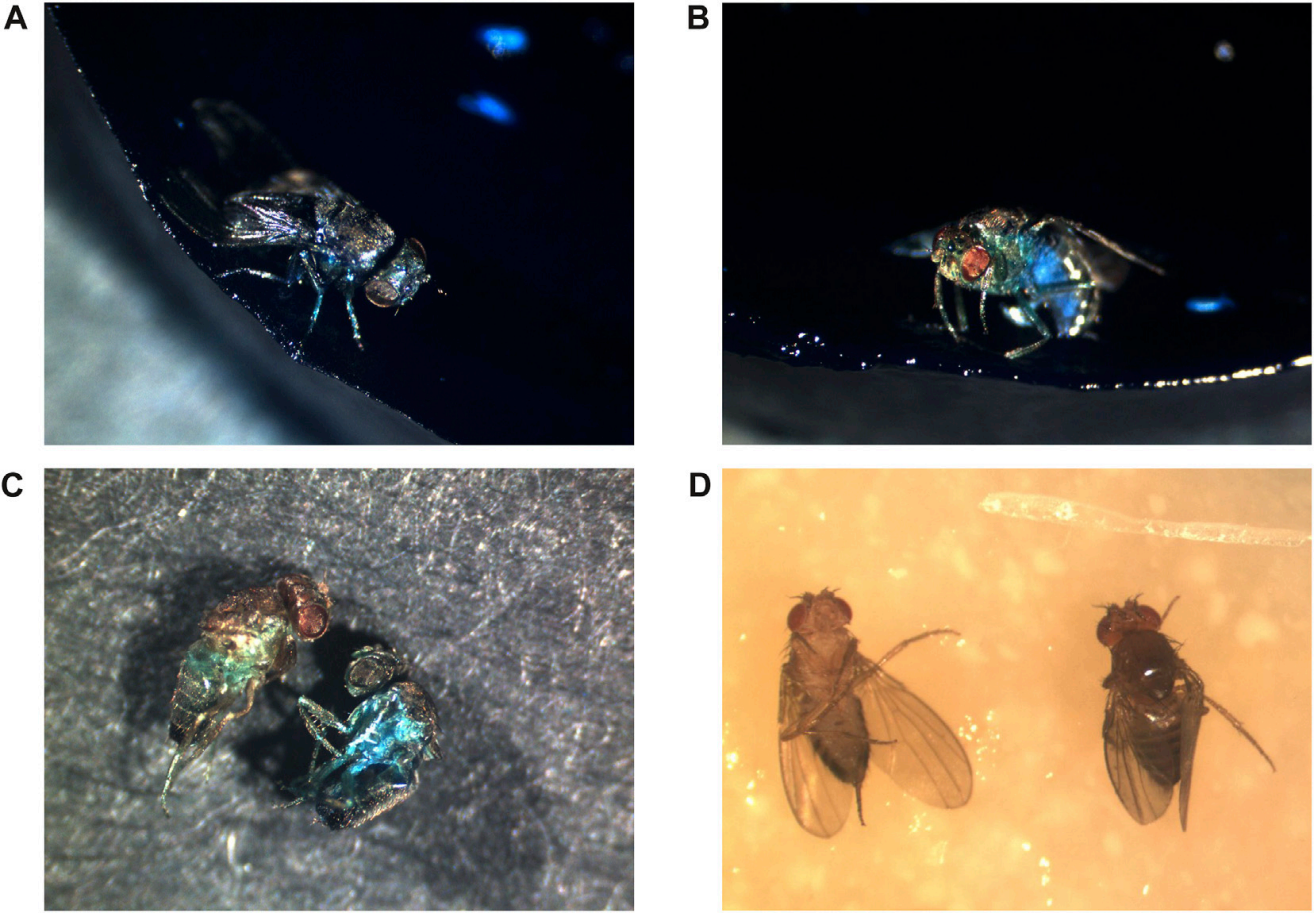
Positions of flies at death. All flies were photographed after death. **(A–C)** *Metchnikowin-GFP* strain flies maintained continuously on food supplemented with 2.5% wt/vol blue dye #1 to reveal potential Smurf phenotype. **(A)** Upright position at death, Smurf phenotype. **(B)** Upright position at death, (no Smurf phenotype). Please note that reflected light from the intensely blue food can produce some light blue color on the image of the fly, as observed on the underside of the abdomen. The lighting was positioned to reduce this effect to the smallest area possible. **(C)** Scoring Smurf phenotype in dead flies. The flies were removed from the media and washed with PBS to remove blue food residue from the surface of the flies, and photographed on black paper. The fly on the left is non-Smurf, recovered from an upright position, the fly on the right is Smurf, recovered from a side position. **(D)** Flies maintained on normal media. The fly on left exhibits supine position at death. The fly on right exhibits upright position at death.

Supine behavior is consistent with a deterioration in nervous system function. Supine behavior can be promoted in young *Drosophila* by mutations that disrupt brain function (Akitake et al., 2015; Sakakibara et al., 2018), as well as by traumatic brain injury (Katzenberger et al., 2013; Lee et al., 2019; Saikumar et al., 2020). Several mutations in mitochondrial genes and ion channel genes result in the bang-sensitive phenotype, where the impact of the fly against a surface causes seizure-like activity, paralysis and supine position (Fergestad et al., 2006). Notably, old wild-type flies often exhibit a bang-sensitive phenotype, and this might be induced by a fall. A fly in supine position cannot access water or food, and even in young flies a lack of water and food results in death within 24–48 h (Schriner et al., 2013; Parkash et al., 2014; Tower et al., 2019). As discussed above, supine position is commonly observed at death for *Drosophila* (see Figure 1D). Here, ∼200 flies were scored for position at death, and this analysis yielded 23% upright, 42% supine, and 35% on side.

In summary, erratic movement, falls and supine behavior are indicative of nervous system deterioration and are predictive of death in *Drosophila*, similar to the #4 unintentional injuries and #7 AD causes of death in humans. These observations are again consistent with the idea that neurodegeneration may be a cause of death in *Drosophila* as well as in humans.

### 3.5 Loss of IBI and the smurf assay

**FIGURE 2:**
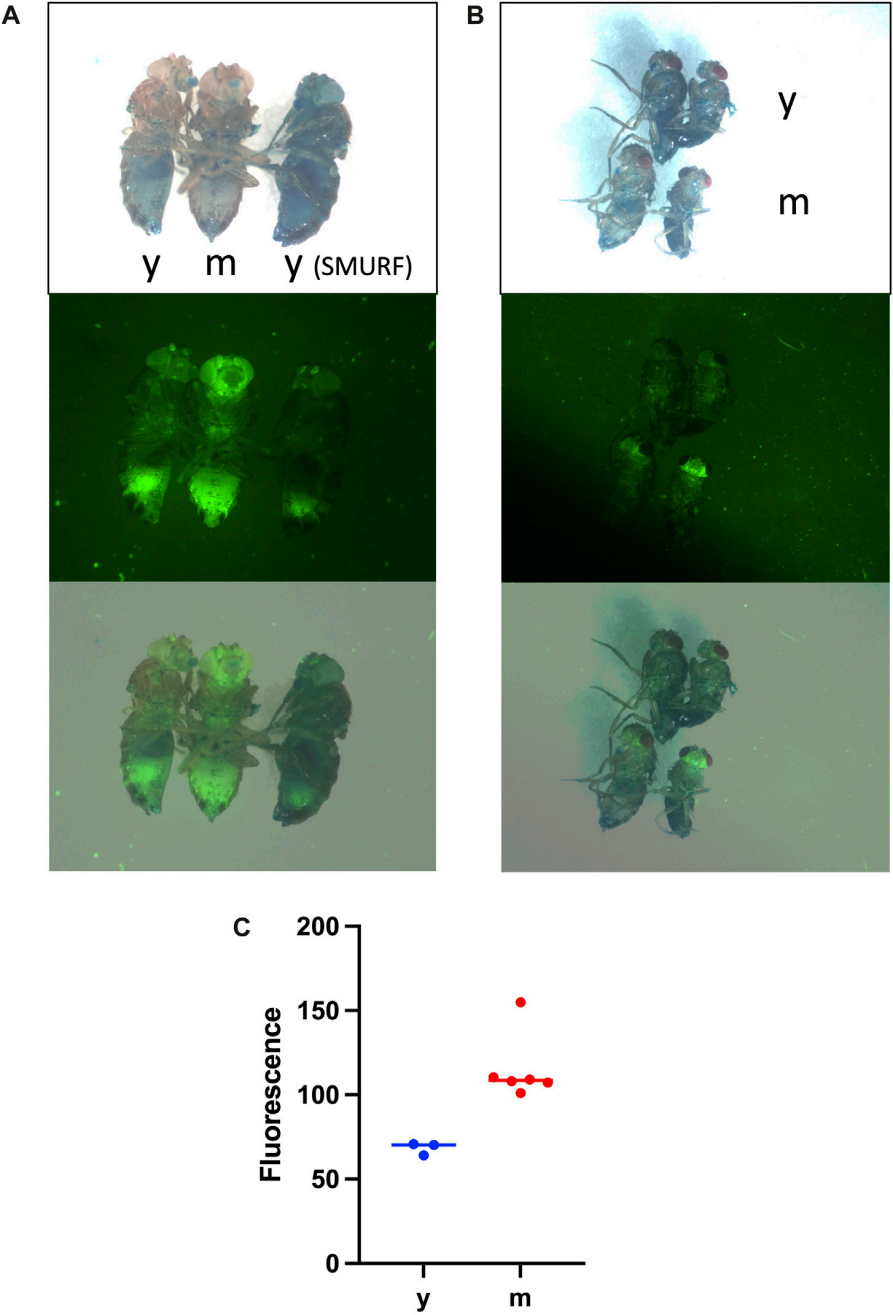
Increased expression of innate immune reporters with age is observed in every fly, in the absence of the Smurf phenotype. Flies were maintained continuously on food supplemented with 2.5% wt/vol blue dye #1 to reveal potential Smurf phenotype. Live flies were anesthetized with CO_2_ gas, wings were removed to facilitate positioning, and flies were imaged on a white flypad surface using Leica MZFLIII fluorescence microscope. Images are visible light (upper panels), GFP fluorescence (middle panels), and visible/GFP overlay (lower panels). **(A)** *Drosomycin*-GFP reporter strain. Three female flies are imaged. y = young, 15 days; m = middle-aged, 35 days. Note increased GFP in the middle-aged fly in the absence of Smurf phenotype; each of 30 additional middle-aged female and male flies scored showed similar increase in GFP in the absence of Smurf phenotype. The female on the right exhibits Smurf phenotype without detectable increase in GFP. **(B)** *Metchnikowin-GFP* reporter strain. Four non-Smurf flies are imaged, with females on the left and males on the right. The upper two flies are a young female and male (y = young, 7 days), and the lower two flies are middle-aged female and male (m = middle-age, 37 days), as indicated. Note increased GFP in the middle-aged flies in the absence of Smurf phenotype; each of 60 additional middle-aged male and female flies scored showed similar increase in GFP in absence of Smurf phenotype. **(C)** Quantification of *Drosomycin*-GFP reporter. Non-Smurf flies were imaged as in **(A)**. y = young, 15 days, n = 3. m = middle-aged, 35 days, n = 6. GFP fluorescence was quantified in the head using Image J. Unpaired, two-sided *t*-test *p* = 0.0057.

Aging-associated deterioration in human IBI, sometimes referred to as “leaky gut”, is implicated in intestinal disease, chronic systemic inflammation, and neurodegeneration (Shemtov et al., 2022). Loss of IBI can also be detected in *Drosophila*, and is associated with increased risk of death. In 2011, Rera, Walker and coworkers introduced the “Smurf” assay, in which *Drosophila* flies are fed a high concentration (2.5% wt/vol) of the non-absorbable blue dye #1 (Rera et al., 2011). In healthy flies, the blue dye is limited to the lumen of the gut, which can be readily observed through the translucent cuticle. However, in a subset of flies, the blue dye is observed throughout all the tissues of the head (including eyes, antennal lobes and proboscis), thorax (including flight muscle and legs), and throughout the abdomen, indicating a loss of IBI and leakage of the dye into the circulatory system (for examples of Smurf, see Figure 1A; Figure 2A). The incidence of the Smurf phenotype was assayed in females, and found to increase with age (Rera et al., 2011), and a follow-up study reported that female Smurf flies had a median life span of 5.5 days or less (Rera et al., 2012). Clark et al. described criteria for scoring a “partial” Smurf, characterized by less intense blue staining of tissues, but still with detectable blue dye in tissues of both the head and the thorax (Clark et al., 2015).

Certain early studies suggested that every female *Drosophila* fly undergoes the transition to Smurf phenotype prior to death (Clark et al., 2015; Tricoire and Rera, 2015; Martins et al., 2018). However, more recent studies reveal that the majority of female and male flies die without ever undergoing Smurf, and that in some strains the Smurf phenotype is completely absent in males. Landis et al. assayed 284 individually-housed female flies continuously throughout their life span for Smurf phenotype, including partial Smurf, with every-other-day scoring of live flies, as well as scoring of dead flies (Landis et al., 2021a). Flies scored as Smurf when alive were dead at the next scoring in every case, and therefore the data indicated that Smurf phenotype robustly predicts death within 48 h or less. Notably, they found that only 40/284 female flies (14%) exhibited Smurf phenotype prior to or at death. In a large-scale and comprehensive study, Bitner et al. assayed 1982 individually-housed male and female flies continuously throughout their life span for Smurf phenotype, including partial Smurf, with every-day scoring of live and dead flies (Bitner et al., 2020). The majority of flies scored as Smurf were dead within 24 h or less. They found that only 22% of males and 34% of females exhibited Smurf phenotype prior to or at death. Therefore, whereas Rera and coworkers recently stated that their data “unequivocally show that every female *Drosophila* dies as a Smurf” (Zane et al., 2023), that conclusion is not consistent with the results from multiple other groups. Finally, Regan et al. reported that Smurf phenotype is completely absent throughout the life span of males of the *wDah* strain, and is present only at very low frequencies in males of the *w[1118]* strain (Regan et al., 2016). In summary, taken together, the data indicate that the Smurf phenotype is a robust biomarker of impending death, but only for a subset of flies.

## 4 Predictive hsp biomarkers of remaining life span in young flies

Certain biomarkers can be assayed in individual young or middle-aged flies, and partly predict subsequent life span. For example, the heat shock protein genes *hsp70* and *hsp22* are induced in response to acute heat stress or oxidative stress, and are also upregulated during normal aging (King and Tower, 1999; Wheeler et al., 1999; Morrow and Tanguay, 2015). *hsp70* encodes a cytoplasmic chaperone, and is upregulated during aging in all tissues, with preferential expression in flight muscle. *hsp22* encodes a mitochondrial chaperone, and is upregulated during aging in all tissues, with preferential expression in nervous system and oenocytes. *hsp22* is also upregulated by mutations that disrupt mitochondrial function and during the mitochondrial unfolded protein response (Fernandez-Ayala et al., 2010; Tower et al., 2014). Transgenic reporters with *hsp70* or *hsp22* promoters driving GFP or DsRED were quantified in individual male flies at young (10 and 20 days) and middle-age (30 days) time points, and were partially predictive of remaining life span (Yang and Tower, 2009). The observation that expression of the genes encoding the cytoplasmic chaperone *hsp70* and the mitochondrial chaperone *hsp22* are both indicative of the individual animal’s remaining life span is consistent with a causative role for proteostasis disruption in *Drosophila* aging and mortality (Moehle et al., 2019; Santra et al., 2019; Francisco et al., 2020). Similarly, proteostasis disruption is causally implicated in each of the top 10 causes of death in humans, including #1 heart disease (Hofmann et al., 2019), #2 cancer (Brancolini and Iuliano, 2020), #3 COVID-19 (Ali et al., 2021), #4 unintentional injury (traumatic brain injury, or TBI) (Saikumar and Bonini, 2021), #5 stroke (Thiebaut et al., 2019), #6 lung disease (Balch et al., 2014), #7 AD (Cozachenco et al., 2023), #8 diabetes (Milardi et al., 2021), #9 liver disease (Baiceanu et al., 2016), and #10 kidney disease (Chen et al., 2021).

## 5 Innate immune response reporter expression in young flies predicts remaining life span

The *Drosophila* innate immune response genes *Drosomycin* and *Metchnikowin* were discovered in the mid-1990s, based on their induction in response to septic injury (Fehlbaum et al., 1994; Levashina et al., 1995). Subsequent studies revealed that *Drosomycin* and *Metchnikowin* are progressively upregulated during normal aging, including in flies where the bacteria have been eliminated using antibiotics or axenic culture conditions (Landis et al., 2004; Ren et al., 2007). Transgenic reporters with the *Drosomycin* or *Mechnikowin* promoters driving GFP were quantified in individual male flies at several time points, at ≤27 days of age, and were found to be partially predictive of remaining life span, both in the absence and presence of antibiotics (Landis et al., 2004).

Because the innate immune response genes are upregulated and are partly predictive of remaining *Drosophila* life span under conditions where bacteria are reduced or eliminated, it suggests they are responding to other age-related signals. Indeed, mitochondrial oxidative stress and mitochondrial DNA and formyl-peptides released from damaged mitochondria are powerful activators of the innate immune response, and are causally implicated in the sterile inflammation, or “inflammaging” that characterizes aging across species, including *Drosophila* and humans (Tower, 2015; Conte et al., 2020). Notably, similar to the results with *Drosophila*, systemic inflammation has emerged as one of the most robust biomarkers of remaining life span in aging humans (Meier et al., 2023).

## 6 Innate immune response reporter increase with age is uncoupled from smurf

As discussed above, the *Drosomycin* and *Mechnikowin* transgenic GFP reporters are progressively upregulated with age in every fly, and are partly predictive of remaining life span when quantified at early time points of ≤27 days. These early time points are before significant death of the cohort begins, and therefore before any significant expression of the Smurf phenotype is expected to occur. However, certain studies have suggested that increased expression of innate immune response genes with age, including increased expression of the *Drosomycin*-GFP reporter, is largely due to flies undergoing the Smurf phenotype (Rera et al., 2012; Martins et al., 2018; Zane et al., 2023). Here, fluorescence imaging experiments were conducted to further demonstrate that increased immune reporter expression during aging occurs independent of the Smurf phenotype.

Groups of young and middle-aged flies were assayed simultaneously for both immune reporter expression and Smurf. The fly strains and culture are as previously described (Landis et al., 2004). For the *Drosomycin-GFP* reporter, 40 young (15 days) flies were compared to 60 middle-aged (35 days) flies. Increased *Drosomycin-GFP* expression was observed in each of the middle-aged flies relative to the young flies, both females (Figure 2A), and males (not shown), in the absence of Smurf. The *Drosomycin-GFP* expression is especially increased in the head, including the eyes, antennae and the brain, and is also increased in thorax and abdomen (Figures 2A, C). Therefore, *Drosomycin-GFP* expression increases with age in every fly, in the absence of Smurf. A rare Smurf fly was present in the young fly group, and this fly did not show evidence of increased expression of *Drosomycin-GFP* (Figure 2A), indicating that Smurf can occur in the absence of reporter activation.

Therefore, whereas it has been suggested in the past that increased expression of the *Drosomycin-GFP* reporter is a “surrogate” for the Smurf phenotype (Martins et al., 2018), that conclusion is not supported by the published or present data.

Similar results were obtained with the *Mechnikowin-GFP* reporter. For *Mechnikowin-GFP* reporter, 40 young (7 days) flies were compared to 60 middle-aged (35 days) flies. Increased expression of the *Mechnikowin-GFP* reporter was observed in each of the middle-aged flies relative to the young flies, both females and males, in the absence of Smurf (Figure 2B). The *Mechnikowin-GFP* reporter was especially increased in the head and brain, and also increased in thorax. A previous study also found preferential expression of the *Mechnikowin-GFP* reporter in the brain, both during normal aging and upon TBI (Swanson et al., 2020).

In summary, expression of both the *Drosomycin-GFP* reporter and the *Mechnikowin-GFP* reporter are increased with age in every fly, in the absence of Smurf phenotype, including at early time points where this expression has been shown to be partly predictive of remaining life span. Moreover, the Smurf phenotype can sometimes occur in young immune reporter flies in the absence of detectable immune reporter induction.

## 7 Tissue interactions

Interactions between tissues are likely to be involved in *Drosophila* death mechanisms. For example, neuronal signals regulate gut physiology and IBI in several ways. (Liu and Jin, 2017). Intestinal fluid homeostasis is regulated by a group of 2–5 neurons that innervate the hindgut, as well as by the neuronal hormone leucokinin (Cognigni et al., 2011). Insulin-like peptides and short neuropeptide F produced by the brain are reported to regulate intestinal stem cell proliferation and maintenance of IBI (Ulgherait et al., 2014; Shen et al., 2016; Chatterjee and Perrimon, 2021). Finally, TBI is reported to cause loss of IBI and Smurf phenotype (Katzenberger et al., 2015a; Katzenberger et al., 2015b). Therefore, neurodegeneration may disrupt gut physiology and contribute to *Drosophila* death through dehydration, starvation and/or loss of IBI.

Signals from the gut in turn regulate brain physiology (Zhao and Karpac, 2020). For example, signals modulated or produced by the gut microbiome are reported to promote maintenance of negative geotaxis activity, reduce neuronal protein aggregates, and increase life span in wild-type flies, AD model flies, and flies with TBI (Westfall et al., 2019; Molina et al., 2021). Consistent with this, disruptions in the human microbiome and loss of IBI are causally implicated in the progression of neurodegenerative disease, including the #7 AD cause of human death (Di Vincenzo et al., 2023; Yadav et al., 2023; Zheng et al., 2023).

Finally, *Drosophila* muscle tissue, and in particular flight muscle, undergoes progressive deterioration during aging associated with mitochondrial malfunction and disrupted proteostasis (Wheeler et al., 1995; Zheng et al., 2005; Demontis et al., 2013; Guo et al., 2023). A recent single-nucleus transcriptomic analysis of *Drosophila* aging confirmed that muscle cells are among the fastest-aging cells, based on increased apoptotic markers, altered gene expression patterns, and loss of nuclei (Jeon et al., 2015; Lu et al., 2023). Indeed, promoting mitochondrial turnover or inhibiting apoptosis in aging muscle has been reported to improve muscle function and increase life span (Si et al., 2019; Kidera et al., 2020). Loss of muscle function during aging might be sufficient to cause falls and/or death of the fly, however, mutations that specifically disrupt muscle function have generally not been associated with supine behavior. Perhaps more likely, loss of muscle function during aging may synergize with neurodegeneration to promote locomotor malfunction and falls, inability of the fly to right itself, and subsequent death.

## 8 Discussion

### 8.1 Multiple mechanisms of death in *Drosophila*

Taken together, the studies discussed above suggest there are at least two mechanisms for death of *Drosophila*, and a possible third. The first mechanism is loss of IBI, as revealed by Smurf assay, which robustly predicts death within 0–∼48 h, but is only detected in a subset of flies. The second mechanism is nervous system malfunction, leading to locomotor malfunction, bouts of erratic behavior, and falls. The aged fly often cannot right itself after a fall to a side-ways or supine position, leading to inability to access the food and subsequent dehydration/starvation. The nervous system malfunction may be sufficient to cause death of the fly, but in the case of a fall to supine or side position, this likely synergizes with the lethal effects of dehydration/starvation. Consistent with the potential importance of nervous system malfunction, it is notable that the age-related increase in innate immune reporter expression is especially abundant in the brain (Figure 2). Finally, some flies die upright without Smurf phenotype (Figures 1B, C), suggesting a possible third mechanism. One possibility is that these flies die from nervous system malfunction without a fall. Alternatively, death might be due to failure of some other essential function or tissue, such as muscle as discussed above, or the Malphigian (renal) tubules (Lang et al., 2019), similar to the #10 kidney disease cause of death in humans. The relative frequency of these death mechanisms appears to vary between strains and culture conditions, which may affect the relative efficacy of various life span interventions.

In the future it may be helpful to further analyze the potential correlations between the different proposed death mechanisms. Death from Smurf and death from loss of nervous system function and falls might be caused by relatively independent mechanisms that proceed at different rates in different flies. Alternatively, there might be a common underlying mechanism, and what cause of death occurs first for a particular individual might be due to specific genetic vulnerabilities and/or be partly stochastic. The complete absence of Smurf in males of the *wDah* strain is consistent with significant sex differences in the relative frequencies of causes of death (Regan et al., 2016). It may be of interest to simultaneously assay Smurf and locomotor behaviors and falls to ask if Smurf occurs at similar frequency in male and female “illderlies” and “wellderlies”. Similarly, it may be of interest to quantify whether Smurf occurs at similar frequency in male and female flies found in upright, side-ways, or supine positions at death.

### 8.2 Mitochondrial maintenance failure as a possible common mechanism

Whether aging results from a single underlying cause, or whether multiple (semi)-independent mechanisms are operating, such as the proposed “Hallmarks” of aging (Lopez-Otin et al., 2023), is an area of significant current interest and research. One appealing model for a single underlying cause that might explain multiple aging phenotypes and “Hallmarks” is the failure in mitochondrial maintenance with age (Tower, 2015; Ventura-Clapier et al., 2017). Mitochondrial maintenance failure is causally implicated in aging-associated proteostasis disruption (Moehle et al., 2019; Ruan et al., 2020). Moreover, mitochondrial malfunction with age is causally implicated in *Drosophila* nervous system malfunction and muscle malfunction, as well as in the induction of the hsp and innate immune response biomarkers of life span discussed above. Mitochondrial malfunction has also been reported to be involved in aging phenotypes in the *Drosophila* midgut (Boumard and Bardin, 2021). For example, disruption of mitochondrial function in the midgut can cause Smurf phenotype and shortened life span (Dai et al., 2020).

PGC1α is a conserved regulator of mitochondrial biogenesis, and conditional expression of PGC1α in adult *Drosophila* midgut using the Gene-Switch system was reported to reduce Smurf incidence and increase life span (Rera et al., 2011). However, another study on life span failed to reproduce the life span extension using the same strains (Landis et al., 2015). This does not rule out a central role for mitochondrial maintenance failure in midgut aging, but does indicate that transgenic upregulation of PGC1α in midgut is not a consistently effective life span intervention. Conceivably, differences in the efficacy of life span interventions, such as PGC1α over-expression in midgut, might be related to differences in the relative frequency of death mechanisms in different studies, as discussed above. Alternatively, or in addition, differences in results between certain studies may be related to the use of the Gene-Switch system. Using Gene-Switch to study aging phenotypes and life span in mated female *Drosophila* is complicated by the fact that the mifepristone drug used to trigger transgene overexpression can itself reduce midgut hypertrophy and extend life span (Landis et al., 2021b).

### 8.3 Potential implications for human aging

As discussed above, the markers and proposed mechanisms for death in *Drosophila* show many similarities to the leading causes of death in humans. Neurodegenerative disease and sarcopenia are both causative in the increased frequency of falls during aging (Yeung et al., 2019; Joza et al., 2020). In United States of America adults age 65 and older, 36 million falls occur each year, and 20% result in serious injury, including hip fracture and TBI (Moreland et al., 2020; Xu et al., 2022). Indeed, falls are the most common cause of TBI-related hospital admissions and deaths. Aging-associated deterioration in IBI, sometimes referred to as “leaky gut”, is implicated in intestinal disease, chronic systemic inflammation, and neurodegeneration (Shemtov et al., 2022). These similarities are consistent with the possibility that conserved mechanisms might underlie aging, and support the further use of *Drosophila* as a model for human aging and aging interventions.

## Data Availability

The original contributions presented in the study are included in the article/Supplementary Material, further inquiries can be directed to the corresponding author.
